# Correction of aging phenotype in the liver

**DOI:** 10.18632/aging.101523

**Published:** 2018-08-10

**Authors:** Katherine Rodriguez, Ashley Cast, Nikolai A. Timchenko

**Affiliations:** 1 Department of Surgery Cincinnati Children’s Hospital Medical Center, Cincinnati, OH 45229, USA

**Keywords:** aging, steatosis, epigenetic, ghrelin, C/EBPa, cdk4

Non-Alcoholic Fatty Liver Disease (NAFLD) is the growing epidemic which is rapidly increasing in USA [1]. The earliest stage of NAFLD, hepatic steatosis (or non-alcoholic fatty liver, NAFL) has no evidence of liver injury, but is characterized by an accumulation of triglycerides in hepatocytes. In some patients, NAFL can progress in age-dependent manner to fibrosis and then to non-alcoholic steatohepatitis (NASH) and cirrhosis. Mechanisms of development of hepatic steatosis are not well understood and approaches to treat hepatic steatosis are not developed. Recent paper by Guillory et al. [2] has investigated the role of the endogenous ligand of growth hormone Ghrelin in development of age-associated hepatic steatosis. The authors clearly demonstrated the deletion of ghrelin prevents development of hepatic steatosis. This prevention is mediated by down-regulation of C/EBPα-p300 axis suggesting that the inhibition of ghrelin activities or C/EBPα-p300 pathway might be considered as a therapeutic approach. In agreement with these findings, Nguyen et al have recently reported that blocking cdk4, a direct activator of C/EBPα-p300 complex, eliminates age-associated hepatic steatosis as well as several age-associated disorders of the liver [3].

Epigenetic alterations play a critical role in the age-associated liver disorders. However, very little is known about age-dependent epigenetic signatures of liver disorders including hepatic steatosis. Epigenetic changes include methylation of CpG islands and changes of chromatin structure by modifications of histones. A recent report identified 152 age-dependent differentially methylated CpG islands between controls and NASH patients. These changes correlated with fibrosis [4]. Another pathway of epigenetic changes, modification of histones in aged livers, has been recently examined in mouse models with modified transcription factor C/EBPα. It has been shown that cyclin dependent kinase cdk4 is elevated in livers of old mice and triggers development of NAFL [3,5]. These investigations raised two important questions. The first question is how is activity of cdk4 up-regulated in aged mice and how does it lead to the accumulation of C/EBPα-p300 complex? The second question is if the activation of cdk4 in old mice is a critical event in epigenetic-dependent development of hepatic steatosis. Two recently published papers shed light on these issues.

In the first paper, Guillory et al investigated age-associated development of hepatic steatosis in mice with deletion of ghrelin, a hormone which promotes adiposity in animals and in humans [6]. At young age, no significant differences were observed. However, while WT mice developed severe steatosis, Ghrelin knockout mice showed significant inhibition of steatosis. Further studies revealed that the enzyme of the last step of synthesis of triglycerides, DGAT1, is not elevated in livers of Ghrelin KO mice, while it is elevated with age in livers of old mice. Searching for molecular mechanisms, Guillory et al have found that activation of DGAT1 promoter does not occur in ghrelin KO mice due to a lack of C/EBPα-p300 complexes. The lack of these complexes is associated with failure of Ghrelin KO mice to phosphorylate C/EBPα at Ser193, the event that is required for the formation of C/EBPα-p300 complexes and further epigenetic activation of promoters of DGAT1 and other enzymes of TG synthesis. This phosphorylation is typically under control of cdk4 and it is likely that the deletion of ghrelin leads to the inhibition of cdk4, suggesting that cdk4 is a key mediator of ghrelin-dependent hepatic steatosis.

In this regard, a recent paper by Nguyen et al examined the role of cdk4 in age-dependent hepatic steatosis using three settings: liver biopsies from old patients with NAFLD, cdk4-resistant C/EBPα-S193A mice and inhibition of cdk4 in old WT mice [3]. These three experimental settings showed that cdk4 is elevated in old patients and degree of elevation correlates with severity of NAFLD. Work with S193A mice and the inhibition of cdk4, revealed that cdk4 is a key driver of the age-associated hepatic steatosis. Surprisingly, the authors found that inhibition of cdk4 not only eliminates hepatic steatosis, but also corrects several other age-dependent liver disorders including cellular senescence [7], heterochromatin structures, E2F1 and RB-dependent pathways of proliferation, liver/body weight ratio and several blood parameters [3]. Figure 1 summarizes studies presented in two recently published reports. These reports suggested that the elevation of cdk4 in livers of old mice and old patients is a key driver of the age-associated liver disorders, and inhibition of cdk4 by FDA-approved inhibitors might be considered as a therapy to correct hepatic steatosis and improve quality of life in elderly.

**Figure 1:**
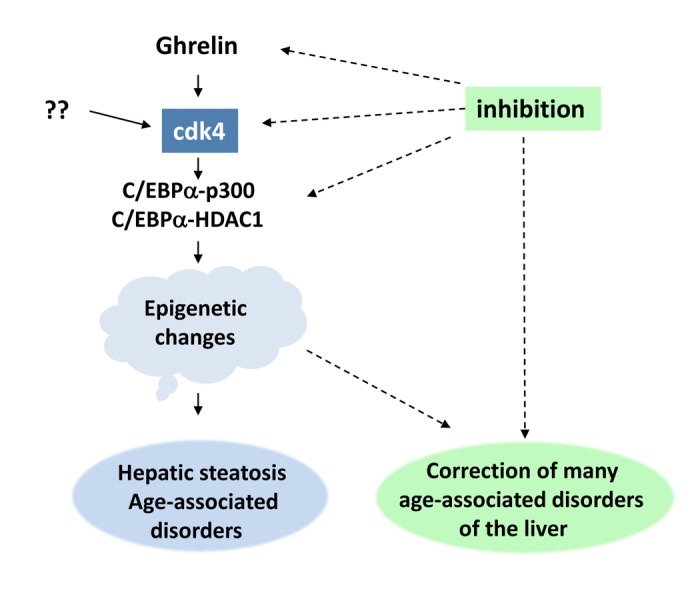
**Ghrelin-cdk4-C/EBPα axis is an essential driver of age-associated liver disorders** (see text).
